# Confocal laser scanning microscopy for rapid optical characterization of graphene

**DOI:** 10.1038/s42005-018-0084-6

**Published:** 2018

**Authors:** Vishal Panchal, Yanfei Yang, Guangjun Cheng, Jiuning Hu, Mattias Kruskopf, Chieh-I. Liu, Albert F. Rigosi, Christos Melios, Angela R. Hight Walker, David B. Newell, Olga Kazakova, Randolph E. Elmquist

**Affiliations:** 1 National Physical Laboratory, Hampton Road, Teddington TW11 0LW, UK.; 2 National Institute of Standards and Technology, Gaithersburg, MD 20899, USA.; 3 Joint Quantum Institute, University of Maryland, College Park, MD 20742, USA.; 4 Graduate Institute of Applied Physics, National Taiwan University, Taipei 106, Taiwan.

## Abstract

Two-dimensional (2D) materials such as graphene have become the focus of extensive research efforts in condensed matter physics. They provide opportunities for both fundamental research and applications across a wide range of industries. Ideally, characterization of graphene requires non-invasive techniques with single-atomic-layer thickness resolution and nanometer lateral resolution. Moreover, commercial application of graphene requires fast and large-area scanning capability. We demonstrate the optimized balance of image resolution and acquisition time of non-invasive confocal laser scanning microscopy (CLSM), rendering it an indispensable tool for rapid analysis of mass-produced graphene. It is powerful for analysis of 1-5 layers of exfoliated graphene on Si/SiO_2_, and allows us to distinguish the interfacial layer and 1-3 layers of epitaxial graphene on SiC substrates. Furthermore, CLSM shows excellent correlation with conventional optical microscopy, atomic force microscopy, Kelvin probe force microscopy, conductive atomic force microscopy, scanning electron microscopy and Raman mapping.

Wafer-scale graphene material is of interest for quantum Hall resistance standards^1–5^ and future nanoelectronics^6, 7^, such as high frequency electronics^8–15^ and photonics^16,17^. Single-domain epitaxial graphene (EG) grown on the silicon face of SiC(0001)^18^ has several advantages, such as removing the need to transfer the graphene onto an insulating substrate for device processing, as is the case for chemical vapor deposition (CVD) growth. Recent progress in CVD and EG growth demonstrates the potential for mass production of homogeneous graphene at the wafer-scale^5,19–22^, and increases the demand for a characterization method that is fast, accurate, and accessible. Previously, optical microscopy was demonstrated to be a useful tool for rapid identification of layer inhomogeneities in EG over hundreds of micrometers^23^. However, low contrast and poor spatial resolution are significant limiting factors. Currently, Raman spectroscopy and scanning probe microscopy (SPM), including Kelvin probe force microscopy (KPFM), are the most widely used methods of characterizing the material quality. Raman spectroscopy is a nondestructive tool for structural analysis of graphene, and is furthermore sensitive to the doping level and strain in graphene^24–29^. For one-layer graphene (1LG) the fingerprint in the Raman spectrum is a symmetric 2D peak at ∼2700 cm^−1^ that can be fitted by a single Lorentzian^26,30^. However, a careful analysis of the shape of the 2D peak is required to identify increasing number of GLs. The topography imaged by atomic force microscopy (AFM) is the another method for determining layer thickness of CVD or exfoliated graphene on various substrates. However, for graphene on SiC, identification of EG layers from the topography is far from straight-forward using AFM alone^31^, due to terrace structure of the SiC substrate, which develops concurrently with the EG and thus has a strong influence on the layer growth and uniformity^5,32^. Recently, KPFM was shown to be a more reliable method for distinguishing the number of EG layers^33^. Nonetheless, Raman and SPM methods are time consuming and typically limit the scan size to a few tens of micrometers. Scaling up the production process requires fast and accurate characterization of the material quality at the wafer scale, while at the same time retaining submicrometer spatial resolution. We believe that confocal laser scanning microscopy (CLSM) is the tool that meets all of the aforementioned requirements with applications in real-time observation of graphene growth^34^ and nanomaterials in biological systems^35,36^.

In this report, we demonstrate that reflection mode CLSM is a superior tool for rapid characterization of large-area graphene and graphene nanostructures on Si/SiO_2_ and SiC, compared to conventional optical microscopy (OM), Raman spectroscopy, AFM, conductive AFM (C-AFM), KPFM, and scanning electron microscope (SEM) methods. CLSM can simultaneously produce intensity and topography images as well as has a lateral resolution that can be pushed beyond the optical diffraction limit. The depth-of-field is enhanced by digitally selecting in-focus regions from multiple images at different focal planes, enabling high resolution over larger areas. First, we discuss CLSM results on various thicknesses of exfoliated graphene transferred to Si/SiO_2_ substrate. By comparing the results to AFM and Raman measurements, we present a method for assessing the CLSM intensity and height for graphene of different thicknesses. Next, we apply CLSM and Raman spectroscopy to CVD-grown graphene transferred to Si/SiO_2_ substrate to demonstrate fast and accurate, largescale analysis. Finally, we apply CLSM to EG on SiC demonstrating the speed, accuracy, and versatility of CLSM compared to OM, SEM, AFM, KPFM, C-AFM, and Raman microscopy.

## Results

### Exfoliated graphene on Si/SiO_2_.

**Fig. 1:**
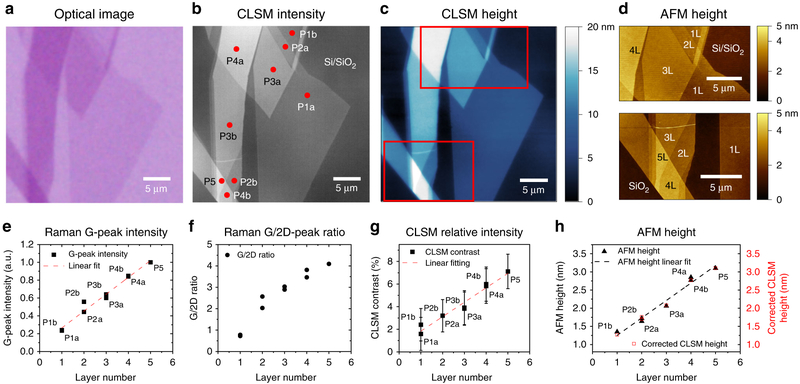
Characterization of exfoliated graphene on Si/SiO_2_ by confocal laser scanning microscopy (CLSM), compared to other methods. **a** Optical microscopy, **b** CLSM intensity, and **c** CLSM height images. **d** Atomic force microscopy (AFM) images of the areas marked in (**c**). **e** G-peak intensity and **f** G/2D-peak intensity ratio. **g** CLSM relative intensity measured at the red points marked in **b**, as a function of the graphene layer thickness. **h** CLSM height measurement corrected with AFM height measurement for 1-5 graphene layers. Raman data were acquired with 514.5 nm excitation. The error bars indicate standard deviation of the measurement

**Fig. 2:**
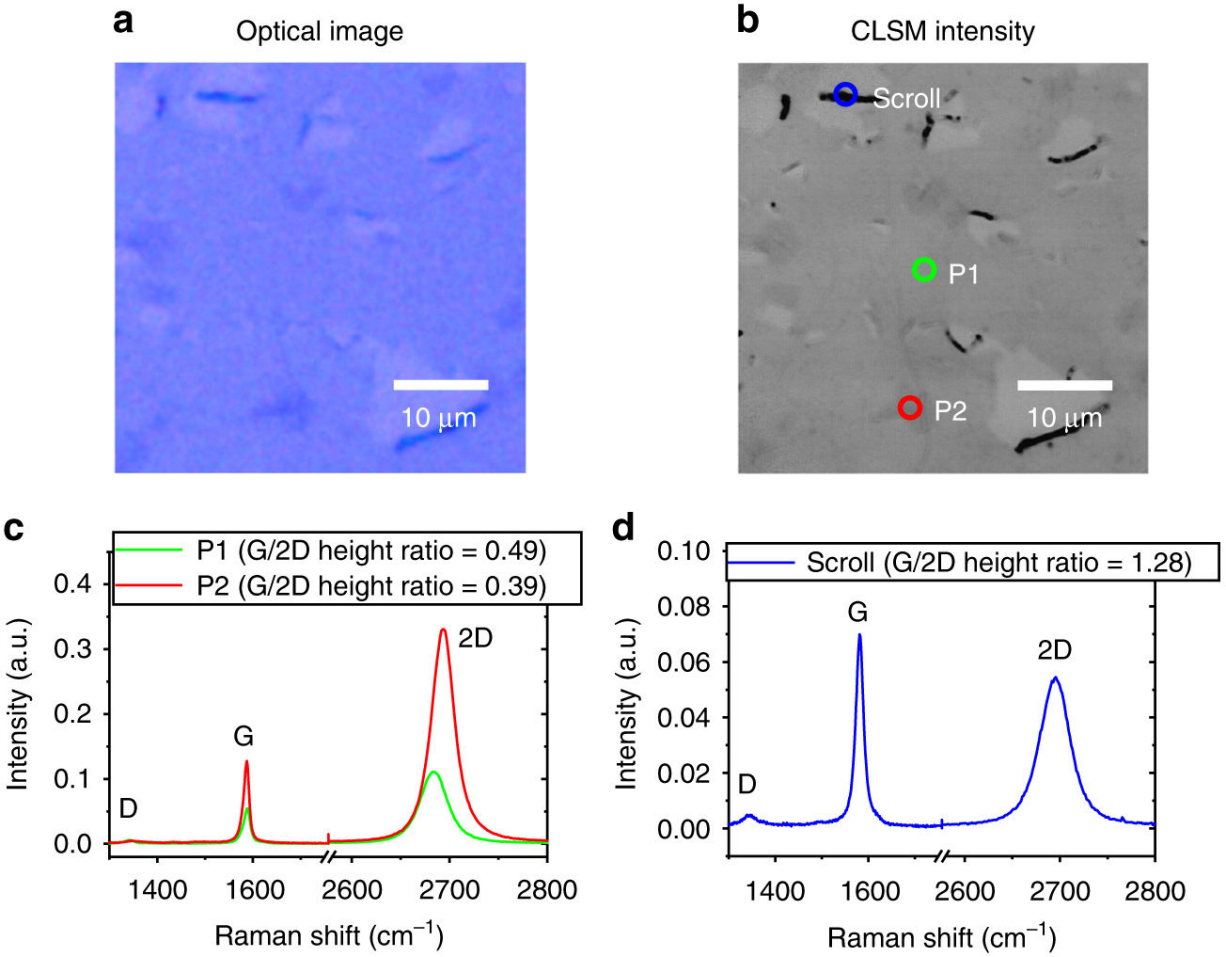
Optical microscopy (OM) and confocal laser scanning microscopy (CLSM) imaging of CVD graphene transferred to Si/SiO_2_ substrate. **a** OM and **b** CLSM intensity images of CVD-grown graphene transferred to Si/SiO_2_ substrate, showing tears and scrolls. **c** Raman spectra for points P1 and P2 in (**b**). **d** Raman spectrum for graphene scroll indicated by a blue circle in (**b**). Raman data was acquired with 514.5 nm excitation

CLSM can simultaneously produce intensity and topography images as well as has a lateral resolution that can be pushed beyond the optical diffraction limit. The depth-of-field is enhanced by digitally selecting in-focus regions from multiple images at different focal planes, enabling high resolution over larger areas. To demonstrate the potential of CLSM for the characterization of graphene, we studied exfoliated graphene transferred to Si/SiO_2_ substrates. The OM images of the sample was carried out on a Nikon L200N optical microscope [see Acknowledgments] in the reflection mode using white light. Figure 1a–c are OM, CLSM intensity, and CLSM height images, respectively, of exfoliated graphene flakes on Si substrate covered by 300 nm of SiO_2_. Both the imaging techniques are performed in reflection mode and compared to the corresponding AFM image in Fig. 1d. Each graphene layer absorbs 2.3% of the incident light^37^. The same region imaged with the CLSM shows a signal-to-noise ratio three times higher than OM as well as provides in situ map of the height. Raman spectra were recorded at the nine different regions of the sample as indicated by red dots in Fig. 1b. For a more in-depth analysis of the layer-dependent optical properties, please see Supplementary Note 1. Analysis of points P1a and P1b matches the description of single LG, where their G-peaks (Fig. 1e) and 2D-peaks can be fitted by a single Lorentzian (FWHM of 26.8 and 28.1 cm^−1^, respectively) and the height ratios of G/2D peaks are ∼0.7 (Fig. 1f). Ni et al.^38^ also reported that the G-peak height increases linearly with the number of layers up to nine layers. This is in good agreement with data shown in Fig. 1e, except for point P2b, where the unexpected behavior may be due to an overlapping of the laser spot with nearby multi-LG domains (P4b and P5). Using the layer numbers determined from the Raman analysis, we found that the CLSM relative intensity (compared to Si/SiO_2_ substrate) also increases approximately linearly with the layer number (Fig. 1g).

The thickness of the first graphene layer (P1b) as measured by AFM is 1.35 ±0.1 nm (Supplementary Table 1), with subsequent layers being measured as 0.48 nm thick (as estimated from the slope of the linear fitting of the raw AFM data in Supplementary Fig. 2). The larger thickness of the first and subsequent graphene layer(s) can be the result of contamination present between the graphene and substrate interfaces^39^. Although the resolution for CLSM height map is ∼10 nm (as specified by the manufacturer), Fig. 1c shows that CLSM is able to distinguish the height of a single layer of graphene. The raw CLSM height values are summarized in Supplementary Table 1 (and Supplementary Note 1), where the first graphene layer is 4.72 ± 0.1 nm, with each subsequent layers being 3.68 nm thick (as estimated from the slope of the linear fitting of the raw CLSM data in Supplementary Fig. 2). Therefore, with proper correction, CLSM can be a fast and reliable method to identify the layer number of exfoliated graphene flakes (Fig. 1h). This is evident by the linearity of the CLSM and theoretical height values.

### CVD graphene on Si/SiO_2_.

Figure 2a, b shows the OM and CLSM images, respectively, of the CVD graphene that has been transferred to Si/SiO_2_ substrate. The CLSM image reveals finer structures that are not clearly visible in the OM image, such as the wrinkle at point labeled as P2 in Fig. 2b, which is about 400 nm wide. Although the Raman spectra from both points P1 and P2 display the features characteristic for single LG, there is a blue shift for the G-peak and a red shift for the 2D-peak at P2, which can be attributed to strain from the wrinkle. The Raman spectra of the scroll shows a more pronounced D-peak due to the curvature effect^40^. Furthermore, the graphene layers in the scroll are not tightly packed, which broadens the 2D-peak. The G/2D peak ratio is also larger due to the presence of several layers of graphene within the scroll.

### EG nanoribbons on SiC.

**Fig. 3:**
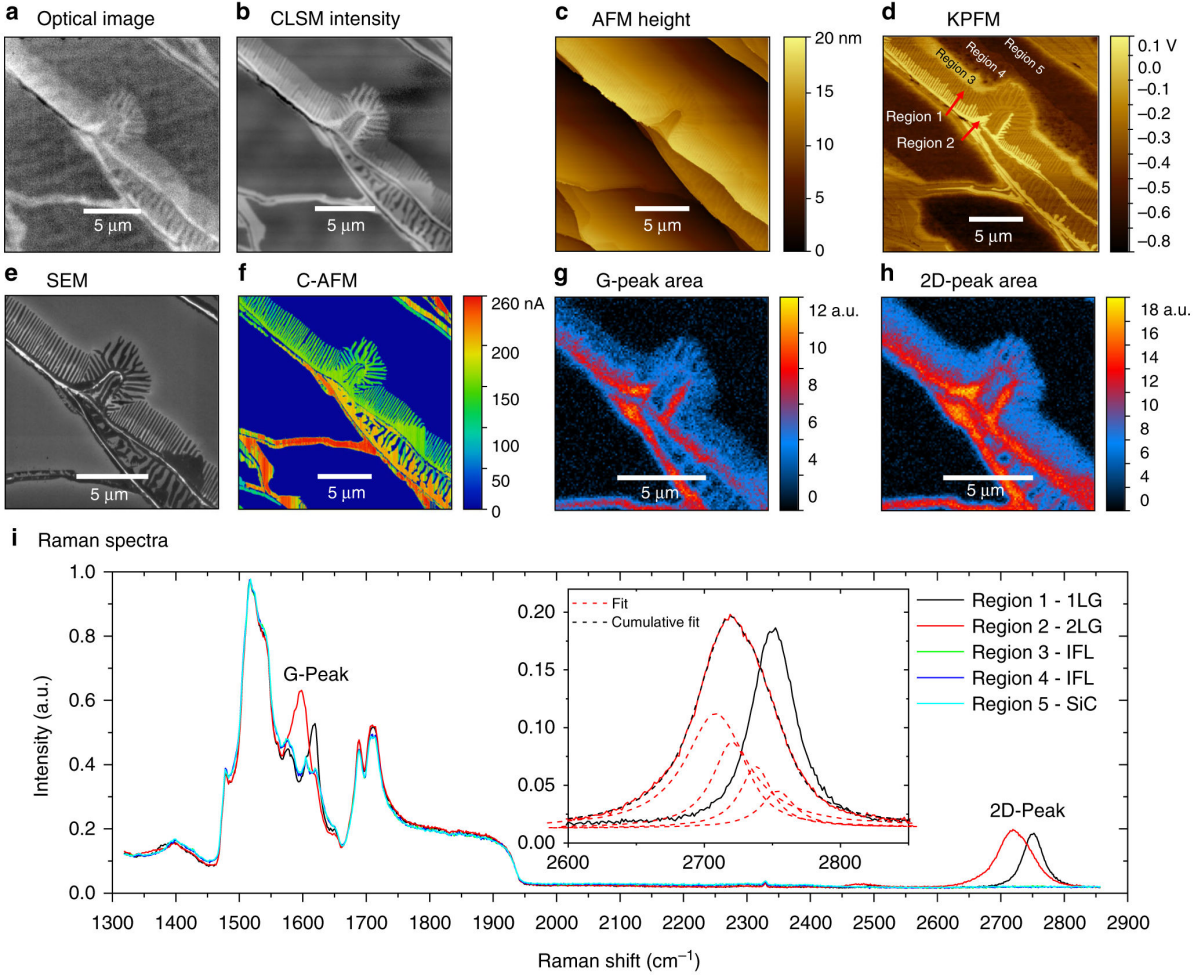
Graphene nanoribbons on SiC characterized by various methods. **a** Optical microscopy (OM), **b** confocal laser scanning microscopy (CLSM) intensity, **c** atomic force microscopy (AFM), **d** Kelvin probe force microscopy (KPFM), and **e** scanning electron microscopy (SEM) with InLens detector, **f** conductive atomic force microscopy (C-AFM) images. **g**-**h** Raman maps obtained for the same area as **a**-**f**, showing the area under the **g** G-peak and **h** 2D-peak. **i** Representative Raman spectra for the five regions indicated in (**d**), where each is an average of 64 individual spectra from the mapped data. Raman data was acquired with 532 nm excitation

We first present the EG sample with partial coverage of graphene, as shown in Fig. 3. The sample is comprised of dense 2D nanoribbons with brighter contrast that are not fully resolved by OM (Fig. 3a), but are more resolved with CLSM (Fig. 3b). Owing to the transparent nature of the SiC substrate, OM imaging is also possible in transmission mode for this particular type of sample (Supplementary Fig. 3), where the contrast is inverted, and the darker regions are associated to graphene with each layer absorbing 2.3% of the incident light^39^.

The darker regions in OM and CLSM intensity images are the electrically insulating interfacial layer or bare SiC, as verified with a several SPM and Raman techniques (Fig. 3c–g). Imaging in the differential interference contrast (DIC) mode produces a threedimensional visualization of the surface morphology with some sacrifice of the graphene contrast (for more details, see Supplementary Note 2 and Supplementary Fig. 3). From comparisons of CLSM, DIC, and scanning probe images such as Fig. 3c, d, we find that the graphene nanoribbons preferentially grow from the step edge toward the adjacent upper terrace. The formation of parallel nanoribbons on SiC(0001) terraces has been attributed to diffusion-limited growth^25^ that may accompany the decomposition of single, SiC atomic layers.

The CLSM intensity image of Fig. 3b shows the remarkable level of detail produced with an acquisition time of only 10 s. Compared to the OM image in reflection mode (Fig. 3a), CLSM provides greatly enhanced lateral resolution and contrast due to the point illumination by a laser source (405 nm wavelength) and by removing the out-of-focus background light with a pinhole at the conjugate focal plane in front of the sensor (i.e., typical confocal configuration). The CLSM intensity image not only shows much higher spatial resolution and fully resolved graphene nanoribbons, but also clearly reveals thin stripes (labeled by red arrows in Fig. 3d) and patches of higher reflectivity along the step edges, indicating two-LG (2LG). Moreover, we determine the lateral resolution of the CLSM to be 150 nm by analyzing the edge spread function from EG images (Supplementary Figs. 4 and Supplementary Table 2, all of which are within Supplementary Note 3).

We obtained additional measurements with AFM, KPFM, SEM, C-AFM, and Raman microscopy, on EG nanoribbons for accurate identification of their structure. The CLSM intensity image in Fig. 3b reveals a dense row of graphene nanoribbons formed from 1LG and 2LG. The KPFM map obtained in situ with the topography (Fig. 3c) reveals substantial variations in the surface potential, with five clear distinct regions (Fig. 3d). Region 1 is assigned to the 1LG nanoribbons which vary in width from ∼100 to 300 nm and lengths of up to a few micrometers. Region 2 is designated as 2LG nanoribbons, which are similar in width to 1LG, but have shorter lengths. Regions 3 and 4 are designated to IFL given that there are no topographical features between them (Fig. 3c), however, the surface potential shows significant differences in the charge between the two regions and is attributed to the close proximity of region 3 to the 1LG nanoribbons. Region 5 is designated as SiC, which is least affected by charge from nearby IFL and graphene.

When SEM imaging is carried out with an InLens detector, the parts of the sample with higher work function lead to a stronger suppression of the backscattered electrons from the surface, resulting in darker contrast in SEM image due to a lower electron intensity sensed by the detector^41^. The SEM is able to clearly distinguish graphene regions from IFL with high spatial resolution (Fig. 3e), but the only indication of 2LG ribbons is faint haloing of IFL regions surrounding them. Additionally, there is no differentiation between IFL and SiC^42^. The C-AFM map is consistent with the designation of regions 3, 4 and 5 where there was no conduction for these electrically insulating regions (Fig. 3f).

Raman spectra of the same region were also collected and fitted to create maps of the G- and 2D-peak area (Fig. 3g, h), position, width and intensity (Supplementary Note 4 and Supplementary Fig. 6). Figure 3i shows the average of 64 representative Raman spectra for each of the five regions indicated in Fig. 3d. The signature single Lorentzian 2D-peak for 1LG is observed for region 1, but broadens significantly and forms the signature shoulder for *ab*-stacked 2LG in region 2 (see fitting of the 2D-peak in the inset of Fig. 3i). These maps clearly show that the coverage of graphene is in excellent agreement with CLSM, AFM, KPFM, and C-AFM.

The 2D forest of graphene nanoribbons is formed in EG samples produced by face-to-graphite growth with reduced process temperatures or reduced growth times (Supplementary Fig. 7a and Supplementary Note 5). In this case, the dense 2D graphene nanoribbon forest along with its conspicuous optical contrast to the IFL patches is a characteristic of incomplete EG coverage. The graphene nanoribbons will eventually merge to form continuous graphene in a succeeding growth (shown in Supplementary Fig. 7b), and the CLSM contrast features will evolve accordingly.

**Fig. 4:**
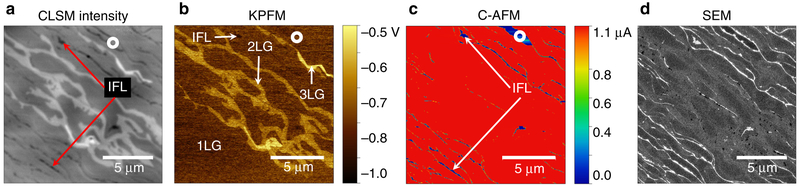
Epitaxial graphene on SiC showing interfacial layer (IFL), single layer graphene (1LG), two-layer graphene (2LG) and three-layer graphene (3LG). (**a**) Confocal laser scanning microscopy (CLSM), (**b**) Kelvin probe force microscopy (KPFM), (**c**) conductive atomic force microscopy (C-AFM) and (**d**) scanning electron microscopy (SEM) image with InLens detector

Next, we investigate samples with full coverage of EG using CLSM, KPFM, C-AFM, and SEM (Fig. 4a–d, respectively). The CLSM intensity image shows that the sample is predominantly covered by 1LG (as conformed by Raman spectra; not shown) and about 10% of the area is covered by narrow patches of bi-(2LG) and tri-LG (3LG) domains (brightest contrast). The higher intensity of the reflected light from thicker graphene layer in the CLSM image is consistent with the linear increase of reflectivity reported by Ivanov et al.^43^, where they measured the power of reflected laser light from the surface of EG on SiC. Comparison of Fig. 4a to the surface potential map (Fig. 4b) of the same region confirms the designation of 1LG, 2LG, and 3LG. However, the darkest lines and patches in Fig. 4a indicated by the red arrows are not as clearly apparent in the KPFM image due to the proximity effect from charging. These features are confirmed to be insulating IFL or SiC by C-AFM, where zero current is measured (Fig. 4c). Moreover, the work function of the sample can be estimated from KPFM by using *Φ*_sample_ = Φ_probe_ − *e*Δ*V*_CPD_, where *Φ*_probe_ is the work function of the probe^33^. Thus, brighter surface potential contrast is associated with lower work function, i.e., work function of 3LG < 2LG < 1LG.

Figure 4d shows the SEM image obtained in a vacuum chamber using the InLens detector to capture backscattered electrons. The IFL/SiC regions appear brightest in the SEM image, which is consistent with the contrast of SEM image for the EG nanoribbon sample (as shown in Fig. 3e and Supplementary Fig. 4b). SEM image of IFL/SiC regions with the brightest contrast has been consistently observed in all the samples presented in this paper and in all the other samples that we have imaged, indicating that IFL/SiC has the lowest work function. 1LG appears the darkest and 2LG has contrast in between the IFL/SiC region and 1LG, indicating work function of IFL/SiC < 2LG < 1LG. Finally, during the SEM imaging, the graphene surface becomes heavily charged by the electron beam and is also exposed to hydrocarbon contamination, both causing deterioration of the image resolution. The dark spots that are tens of nanometers in size, which only appear in the SEM image that was obtained last, are likely to be the hydrocarbon contamination. In contrast to that, CLSM is noninvasive and thus does not influence the imaging quality over time.

## Discussion

In this paper, we demonstrate CLSM, a fast and nondestructive characterization method for optical imaging of graphene, which produces images of optical intensity and height in ambient air, without any prior sample preparation. We would like to stress that of course CLSM is not the only technique to study graphene and should be used in conjunction with other methods for determination of required physical properties. While CLSM cannot determine all physical parameters for any given sample, it can distinguish features such as thickness inhomogeneity, folds, tearing, and nanoribbons of graphene on various substrates with high spatial resolution (150 nm).

For graphene on Si/SiO_2_ substrate, CLSM images show excellent correlation to OM, Raman spectroscopy, and AFM height mapping, where the latter two can be used to calibrate the CLSM intensity and height to directly determine graphene layer thickness. For graphene on SiC substrate, the measured reflected intensity from 1LG is ≈3% higher than that from adjacent IFL regions and the reflected intensity from 2LG is ≈2% higher than the 1LG (see Supplementary Note 6). Through direct correlation to the results from Raman spectroscopy, SEM, and scanning probe microscope including AFM, C-AFM, KPFM, CLSM imaging reveals that EG starts to form at the edges of SiC terraces as parallel graphene nanoribbons. The nanoribbons then merge into a continuous, uniform mono-LG under proper processing conditions. Micrometer-sized, bilayer and few LG patches are found as high contrast regions in the CLSM images of overgrown samples. Compared to the complementary methods used in this paper, CLSM not only has a much higher throughput for detecting such regions, it is also insusceptible to surface contamination or surface charging, which will strongly affect the resolution and even the contrast of other imaging techniques such as KPFM or SEM. Although Raman spectroscopy also has advantages as a nonintrusive optical method, with the modern micro Raman systems having laser spot sizes on the order of hundreds of nm corresponding to the diffraction limit, CLSM can produce a map with similar resolution, simultaneously being able to image much larger areas in a much shorter time frame, which is a significant advantage.

We propose that high spatial resolution CLSM images can provide inspection of wafer-scale graphene, selection of material and locations for more efficient fabrication (see Supplementary Figs. 9–11 within Supplementary Note 7), as well as analysis of device quality and failure modes (Supplementary Fig. 12 within Supplementary Note 8).

## Methods

### Confocal laser scanning microscopy.

CLSM was performed using an Olympus LEXT OLS4100 system [see Acknowledgments] fitted with ×5, ×10, ×20, ×50, and ×100 objectives (numerical apertures: 0.15, 0.30, 0.60, 0.95, and 0.95, respectively) and with up to ×8 further optical zoom. This enables the CLSM to image areas with field of view ranging from 2560 to 16μm, which translates to total magnification range from ×108 to ×17,280. The system employs a 405 nm wavelength violet semiconductor laser, which is scanned in the *X–Y*directions by an electromagnetic micro-electro-mechanical systems scanner and a high-precision Galvano mirror, and a photomultiplier to capture the reflected light and generate images up to 4096 × 4096 pixels with horizontal spatial resolution of 150 nm. The confocal optical setup only allows the reflected light that is in-focus to pass through the circular confocal pinhole, thus eliminating flare from out-of-focus regions, but resulting in a very shallow depth of field. To increase the focus resolving capability, a series of images along Z-axis are taken around the median focus height, with separations as small as 60 nm. For each pixel, an ideal Intensity-Z curve is calculated to fit the intensities in these images and extract the maximum value, which in turn is used to create a final 2D intensity image. The system is operated in ambient air and does not require any sample preparation for clean samples.

### Atomic force microscopy.

AFM was performed in tapping-mode in air using a Bruker Dimension FastScan SPM. In this mode, the probe oscillates at its fundamental resonance (*f*_0_) and a feedback loop tracks the surface of the sample by adjusting the Z-piezo height to maintain a constant amplitude of the cantilever oscillation. The phase of the cantilever oscillation is also compared to the sine wave driving the cantilever oscillation, and thus, AFM achieves simultaneous mapping of the topography and tapping phase, which is a measure of the energy dissipation between the probe and sample, thus encompassing variations in adhesion, composition, friction, viscoelasticity, and other mechanical properties of the sample^43^.

### Conductive atomic force microscopy.

C-AFM was performed using a Bruker Dimension Icon SPM by raster scanning a Pt probe across the sample surface. The C-AFM scans were performed with 250 mV bias voltage applied to the sample and the resulting current flowing through the probe at each pixel of the scan area was measured by a current amplifier. EG’s high electrical conductivity and good adhesion allow precise mapping of the nanostructures by C-AFM unless they are isolated by nonconducting SiC or interfacial layer carbon.

### Kelvin probe force microscopy.

KPFM was performed by means of frequency modulation (FM) using a Bruker Dimension Icon SPM^37^. During FM-KPFM, the surface of the sample is tracked and measured using the AFM feedback method described above. Additionally, a low frequency (*f*_mod_), AC voltage (*V*_AC_) is applied to the electrically conductive probe, which shifts the cantilever resonance due to the electrostatic attraction/repulsion and thus produces side lobes at *f*_0_±*f*_mod_. When the FM-KPFM feedback loop applies an additional DC voltage to the probe (*V*_DC_), the amplitude of the side lobes is proportional to the difference between *V*_DC_ and the surface potential of the sample (also referred to as the contact potential difference, *V*_CPD_). The surface potential is determined by the *V*_DC_ minimizing the side lobes, i.e., when potential of the probe is equal to the potential of the sample. The surface potential map is obtained by recording *V*_DC_ pixel by pixel. The surface potential values of the sample can be converted to a work function using, *Φ*_Sample_ = *Φ*_probe_ − *e*Δ*V*_CPD_, provided the work function of the probe (Φ_probe_) is known. For further details see ref. ^44^

### Raman spectroscopy.

Raman spectra of graphene on Si/SiO_2_ were acquired under ambient conditions with 514.5 nm excitation (Renishaw InVia), which is focused to an approximately 1 μm spot on the sample through a ×50 objective (0.75 NA). The Raman spectra and mapping of EG on SiC were acquired under ambient conditions with 532 nm excitation (Renishaw InVia), which is focused to an approximately 0.8μm spot on the samples through a ×100 objective (0.85 NA). Raman maps were performed by raster scanning the laser with a step size of 100 nm and collecting the spectra with an exposure time of 1 s for each point, 1800l/mm grating and high confocality (20μm slit opening). Raman maps of the G-and 2D-peaks area, intensity, width and shift were generated from fitting the data.

## Data availability

The datasets generated and analyzed during the current study are available from the corresponding author on request.

## Supplementary Material

Supp1
