# Supplementary material for: Confocal laser scanning microscopy for rapid optical characterization of graphene
Source: Commun Phys. Author manuscript; Available in PMC 2019 May 13. (PMC6512973; doi:10.1038/s42005-018-0084-6)
Supplement: Supp1 [file NIHMS1520842-supplement-Supp1.pdf]

Supplementary Information

Supplementary Note 1. CLSM CHARACTERIZATION OF EXFOLIATED GRAPHENE ON Si/SiO$_2$ SUBSTRATE

Supplementary Figures 1a and 1b are the conventional optical and CLSM height images, respectively, of the same exfoliated graphene flake as shown in Figure 1 in the main article. The inset of Supplementary Figure 1b is the AFM topography image of region marked by yellow box in Supplementary Figure 1b. Supplementary Figure 1c is the Raman spectra for all nine spots marked in Figure 1b in main article, where the G-peak height increases with the layer number. Supplementary Figure 1d shows the CLSM intensity and height profiles for the cross section indicated by the red dashed line in Supplementary Figure 1b, showing that both properties increase linearly with the layer number. Supplementary Figure 1e compares the CLSM height profile to the AFM height profile for the same cross section. While the AFM measurement of the first graphene layer is strongly affected by the gap between the substrate and the graphene flake, the CLSM intensity and height measurements are not so susceptible to this problem.

In Supplementary Figure 2, we applied linear fitting for both CLSM and AFM height measurement as a function of the layer number and obtained the following equations:

$$T_{g-CLSM} = 3.6767 \times H_{CLSM} + 0.8986 \quad \text{(S1)}$$

$$T_{g-AFM} = 0.4800 \times H_{AFM} + 0.7728 \quad \text{(S2)}$$

Where T_g is the thickness of graphene and H is the height measured by either CLSM or AFM. The data of graphene height plotted in Supplementary Figure 2 are extracted from Figures 1b-1d in the main article using the "statistical quantities" tool in Gwyddion[1], corresponding to the 7 spots listed in Supplementary Table 1. From the above equations, we can calibrate the CLSM height measurement as

$$T_{g-CLSM-cal} = \frac{0.4800}{3.6767} \times T_{g-CLSM} + \left[0.7728 - \left(\frac{0.4800}{3.6767} \times 0.8986\right)\right] \tag{3}$$

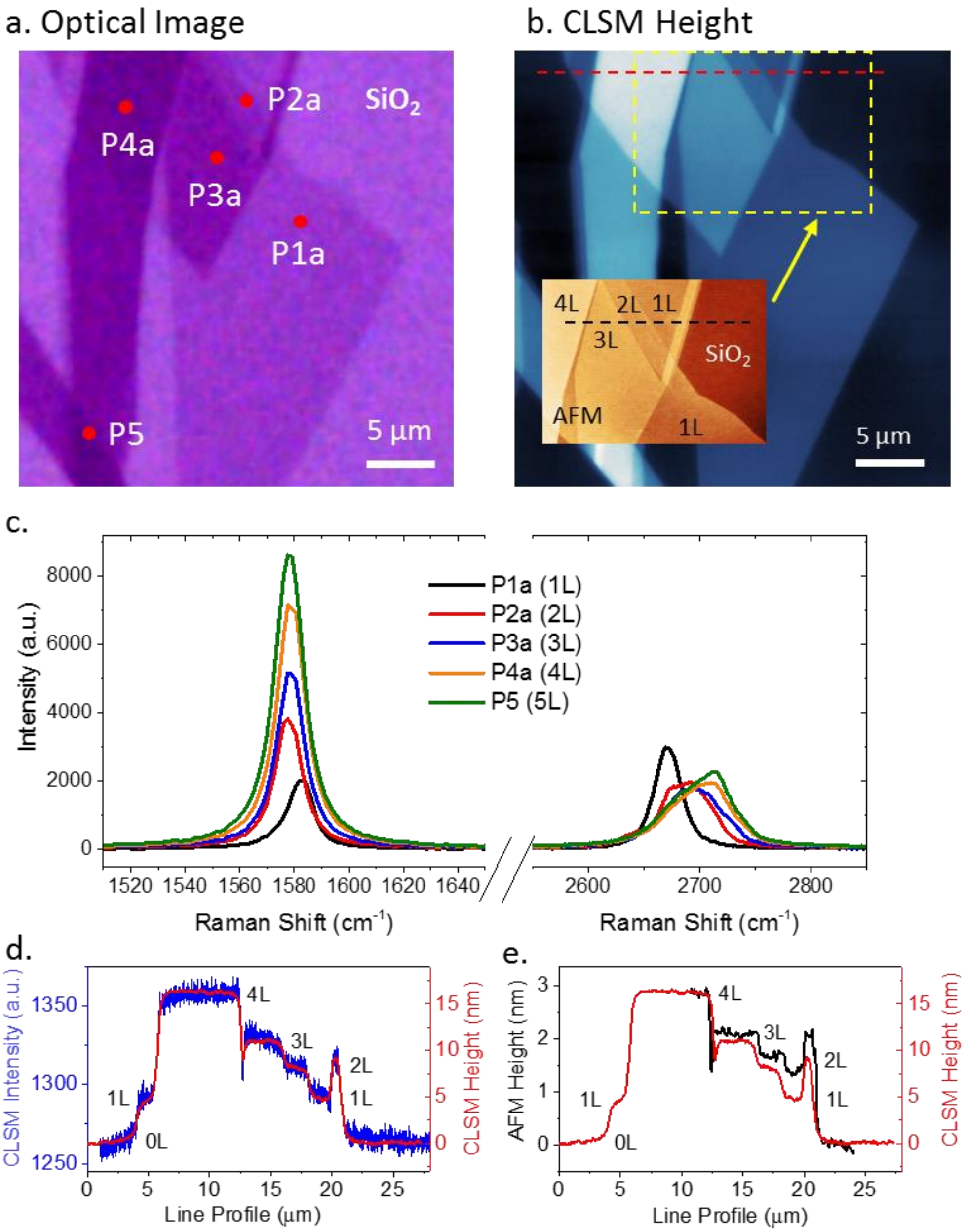

Supplementary Figure 1. (a) Optical image of the same exfoliated graphene flake as discussed in Figure 1 in the main article. (b) CLSM height image of the same flake as in (a). (c) The Raman spectra of 1-5 layer graphene marked as in Figure 1b in the main article showing the evolution of G- and 2D-peaks with the layer number. (d) The CLSM intensity (blue) and CLSM height (red) profiles along the red dashed line shown in (b). (e) The AFM height (black) and

CLSM height (red) line profiles along the black and red dashed lines shown (b). Raman data was acquired with 514.5 nm excitation.

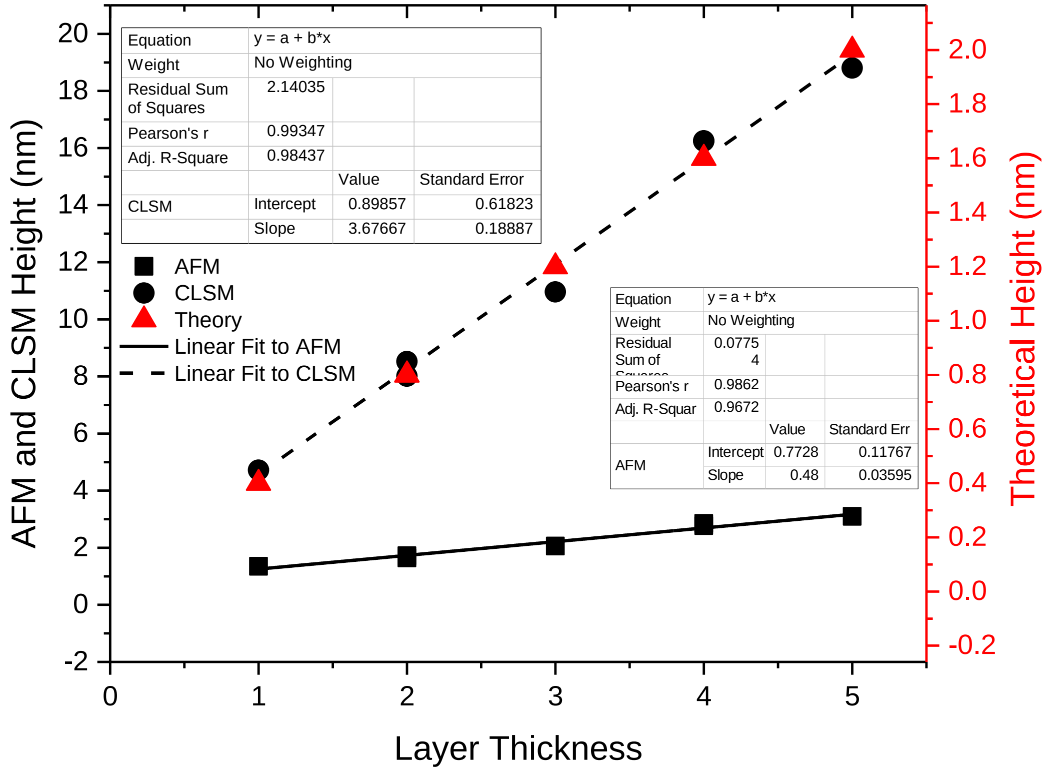

Supplementary Figure 2. Black solid line is linear fitting to the AFM height data points (black squares) and black dashed line is linear fitting to the CLSM height data points (black circles), both as a function of the layer numbers.

Supplementary Table 1. Summary of the AFM height and CLSM height data used in Figure 1 in the main article. The error represent the standard uncertainties of the measurement.

**Points**	**AFM height (nm)**	**Error (nm)**	**CLSM height (nm)**	**Error (nm)**
P1b	1.35	0.10	4.72	0.11
P2a	1.64	0.11	8.01	0.20

P2b	1.72	0.13	8.52	0.13
P3a	2.06	0.10	10.96	0.11
P4a	2.85	0.16	16.24	0.12
P4b	2.84	0.11	16.25	0.16
P5	3.11	0.11	18.55	0.12

Supplementary Note 2. CONVENTIONAL OPTICAL AND CLSM INTENSITY IMAGES OF EPITAXIAL GRAPHENE NANORIBBONS

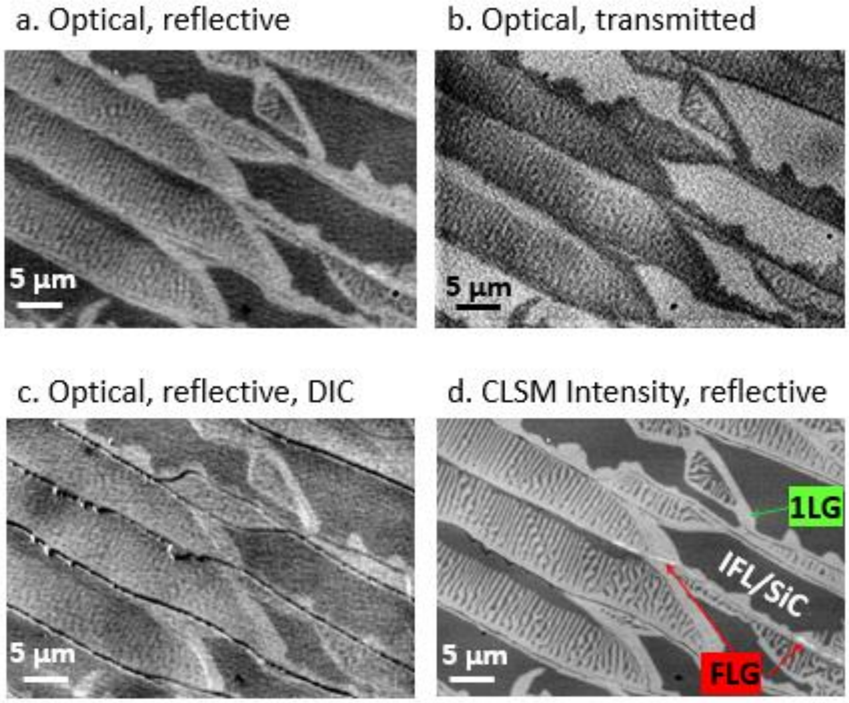

Supplementary Figure 3. Optical images obtained in (a) reflection, (b) transmission, (c) reflection with differential interference contrast (DIC) and CLSM intensity in reflection modes.

The conventional optical images of epitaxial graphene on SiC in Supplementary Figure 3 were obtained with a Nikon Eclipse L200N optical microscope, which is capable of operating in the reflection, transmission or differential interference contrast (DIC) mode using white light. The brighter regions of the reflective optical image are associated to graphene which is partially covering the surface[2], and darker regions are insulating interfacial layer (IFL) or bare SiC, as

verified later with a host of scanning probe microscopy and Raman techniques. Supplementary Figure 3b shows the transmission image of the same area as Supplementary Figure 3a, where the contrast is now inverted. Imaging in the DIC mode produces a 3-dimensional visualization of the surface morphology with certain sacrifice of the graphene contrast. Supplementary Figure 3d is the CLSM image of the same area as in Supplementary Figures 3a-c, which not only shows a much higher special resolution, but also clearly reveals thin stripes (labeled by red arrows) and patches of higher reflectivity along the step edges, indicating few-layer graphene (FLG).

Supplementary Note 3. ESTIMATION OF LATERAL RESOLUTION FOR CLSM IMAGE

Supplementary Figure 4a-b shows the analysis of a CLSM and SEM image containing dense forest of 2D graphene nanoribbons by using the image processing program ImageJ. The red curve in Supplementary Figure 4c is the averaged profile crossing the graphene nanoribbons marked by the red rectangular box, compared to the averaged profile (blue curve) from the same region of the SEM image. For the nineteen dips clearly seen from the SEM profile, seventeen corresponding peaks (counted from right) can be distinguished from the CLSM profile for the graphene nanoribbons with width varying approximately from 120 nm to 230 nm.

We further estimated the lateral resolution of the CLSM image in Supplementary Figure 4a by analyzing the edge spread function (ESF). The blue points in Supplementary Figure 4d-e are the averaged profile across the edges marked by two red filled boxes in Supplementary Figure 4a. An integrated Gauss function is used to fit the averaged profile, with the high plateau on the left defined as 100% brightness and the low plateau on the right defined as 0% brightness. The lateral resolution is estimated by calculating the edge width between two reference points with 20% and 80% of brightness (Fit 20/80). The inset label in Supplementary Figures 4d-e shows that the lateral resolution is approximately 149 nm and 161 nm for the red filled boxes marked 1

and 2 in Supplementary Figure 4a, respectively. The lateral resolution will be affected by factors such as the contrast level and materials, and therefore varies from sample to sample, or from region to region on the same sample.

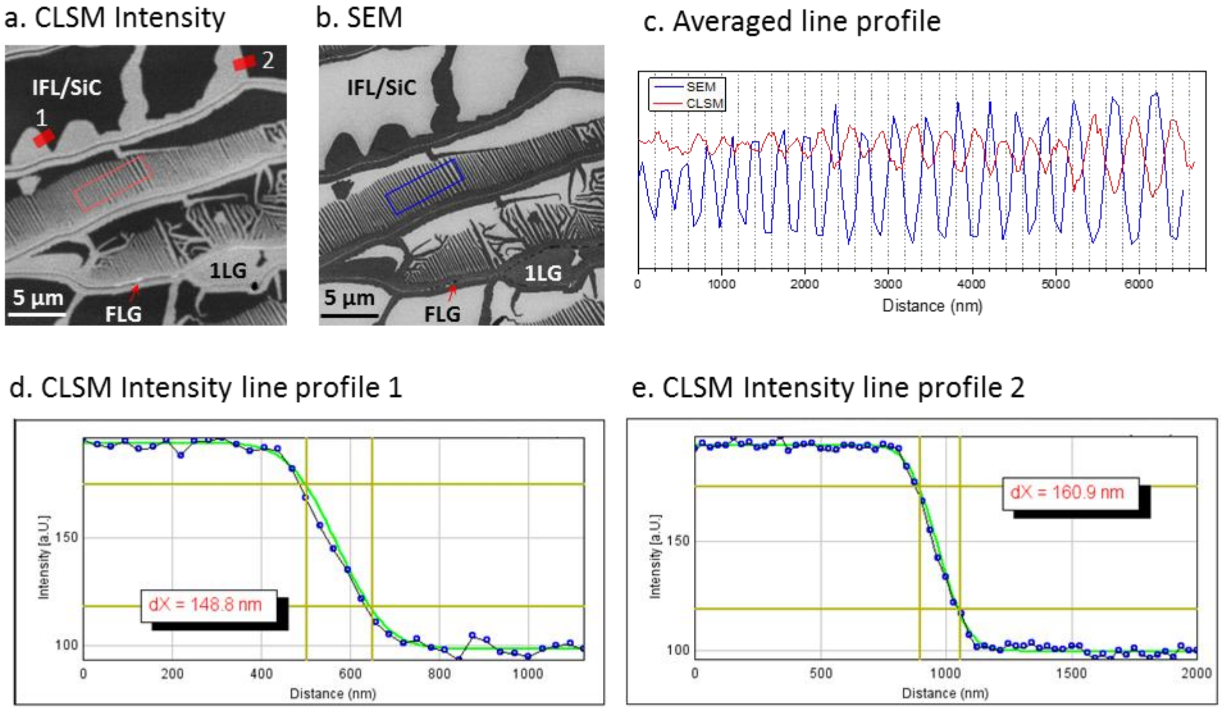

Supplementary Figure 4. Analysis of the lateral resolution of the CLSM and SEM EG images by ImageJ. (a) CLSM and (b) SEM images of graphene nanoribbons on SiC. (c) Averaged profile for the red and blue rectangular boxes in (a-b). (d) and (e) CLSM intensity profiles at the edges of graphene and IFL/SiC indicated by the red filled boxes marked 1 and 2 in (a), with Gauss simulation (green line).

Supplementary Figure 5 shows the CLSM image of an epitaxial graphene sample containing regions of IFL, 1LG 2LG, and 3LG. The lateral resolution values estimated from the CLSM image in Supplementary Figure 5 show that the 20/80% of ESF for the edges denoted in the Supplementary Table 2 vary from approximately 97.3 nm to 185.8 nm. Based on analysis of

more than 10 CLSM images from different samples, we estimate that the lateral resolution of our CLSM images is approximately 150 nm.

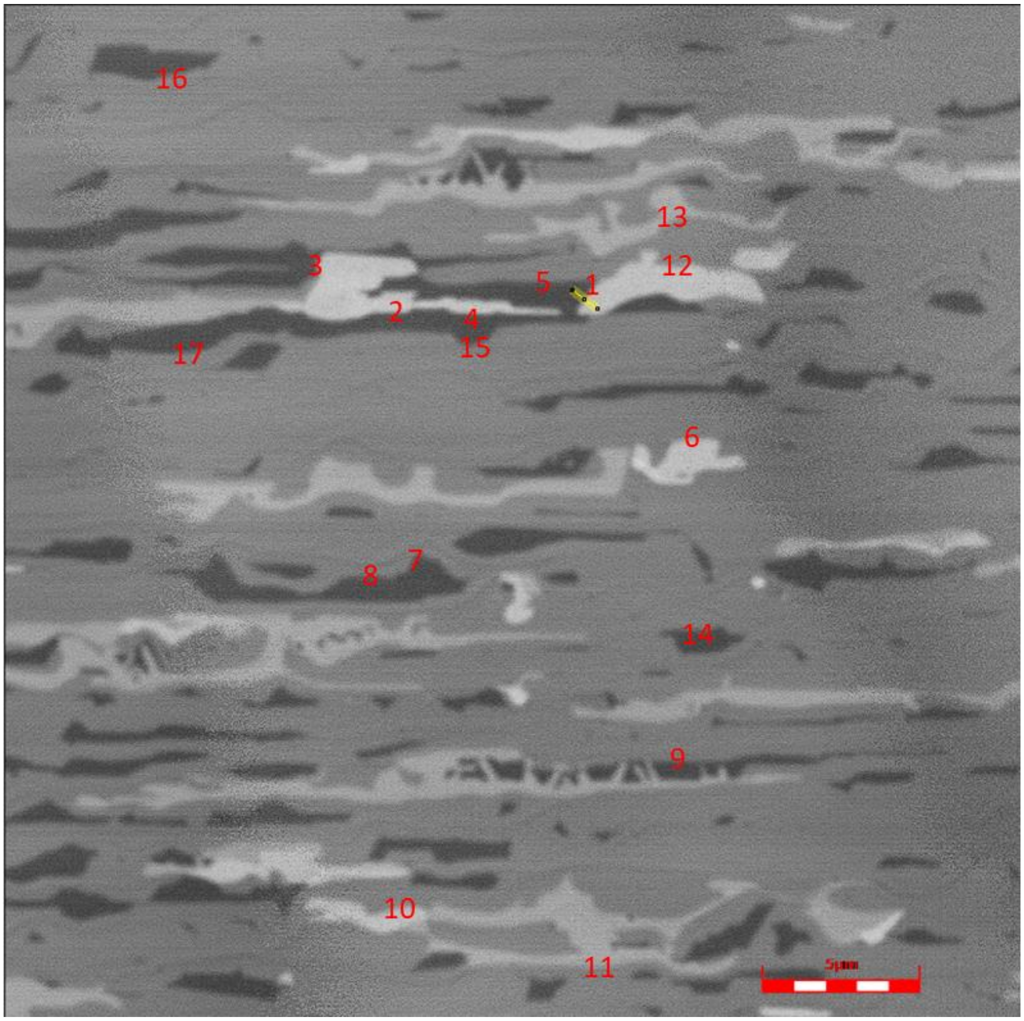

Supplementary Figure 5. Example of large-area coverage and its corresponding brightness. CLSM image of EG sample covered by dominant single layer graphene (1LG). The lowest brightness indicates IFL region. Higher brightness corresponds to thicker graphene layer.

Supplementary Table 2. Summary of the edge widths. A table with edge width calculated from "Fit 20/80" algorithm, for the locations indicated in Supplementary Figure 5.

Edge Index	20/80 Fitting Width (nm)	Error (%)
1	139	6.7

2	105	5.5
3	158	6.4
4	97.3	8.5
5	144	11.7
6	136	7.1
7	161.8	21.1
8	149.4	23.7
9	142	12.8
10	148	14.3
11	132	26.5
12	124.7	16.2
13	123.8	26.7
14	153.9	24
15	185.8	34.3
16	110.5	25.6
17	163.1	19.6

Supplementary Note 4. RAMAN MAPS FOR EPITAXIAL GRAPHENE NANORIBBONS

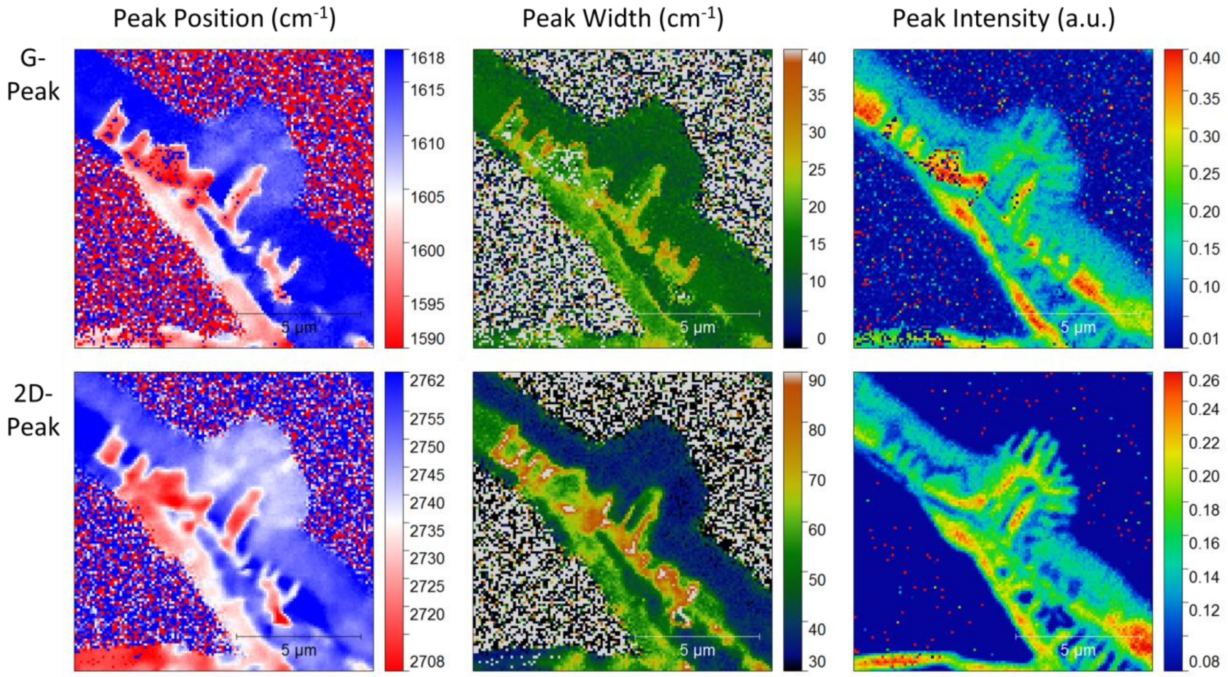

Supplementary Figure 6. Raman maps of the peak position, width and intensity for the G- and 2D-peaks for the epitaxial graphene nanoribbons area presented in Figure 3 of the main article. Raman data was acquired with 532 nm excitation.

Supplementary Figure 6 is a summary of the Raman analysis performed on epitaxial graphene nanoribbons to provide a comparison with the optical imaging techniques presented in the main article.

Supplementary Note 5. EVOLUTION OF THE OPTICAL CONTRAST FEATURES FROM INCOMPLETE EG TO CONTINUOUS EG

Supplementary Figure 7a is a reflective optical image of a face-to-graphite sample obtained by Nikon Eclipse L200N optical microscope with a 50× objective, showing incomplete single layer graphene (1LG) coverage. The graphene nanoribbons merged into continuous graphene in a succeeding growth, as shown in Supplementary Figure 2b. The conspicuous contrast from the interfacial layer regions (the darker contrast in Supplementary Figure 2a) disappeared in Supplementary Figure 2b. Instead, only narrow lines of higher brightness are seen after the second growth along the step edges, indicating few layer graphene, as confirmed by Raman spectroscopy. Supplementary Figure 7c and 7d are cropped from Supplementary Figure 7a and 7b, respectively, showing the same region where a Raman map (Supplementary Figure 7e) has been generated after the second growth. The spectrum from a spot on the terrace, marked by a green circle in Supplementary Figure 7e, show a symmetric 2D-peak (the green curve in Supplementary Figure 7f) that can be fit by a single Lorentzian (the black dashed line in Supplementary Figure 7f), confirming the existence of 1LG. The spectrum from the spot at the step edge, marked by a red circle in Supplementary Figure 7e, shows a much wider asymmetric 2D-peak (the red curve in Supplementary Figure 7f), indicating few layer graphene.

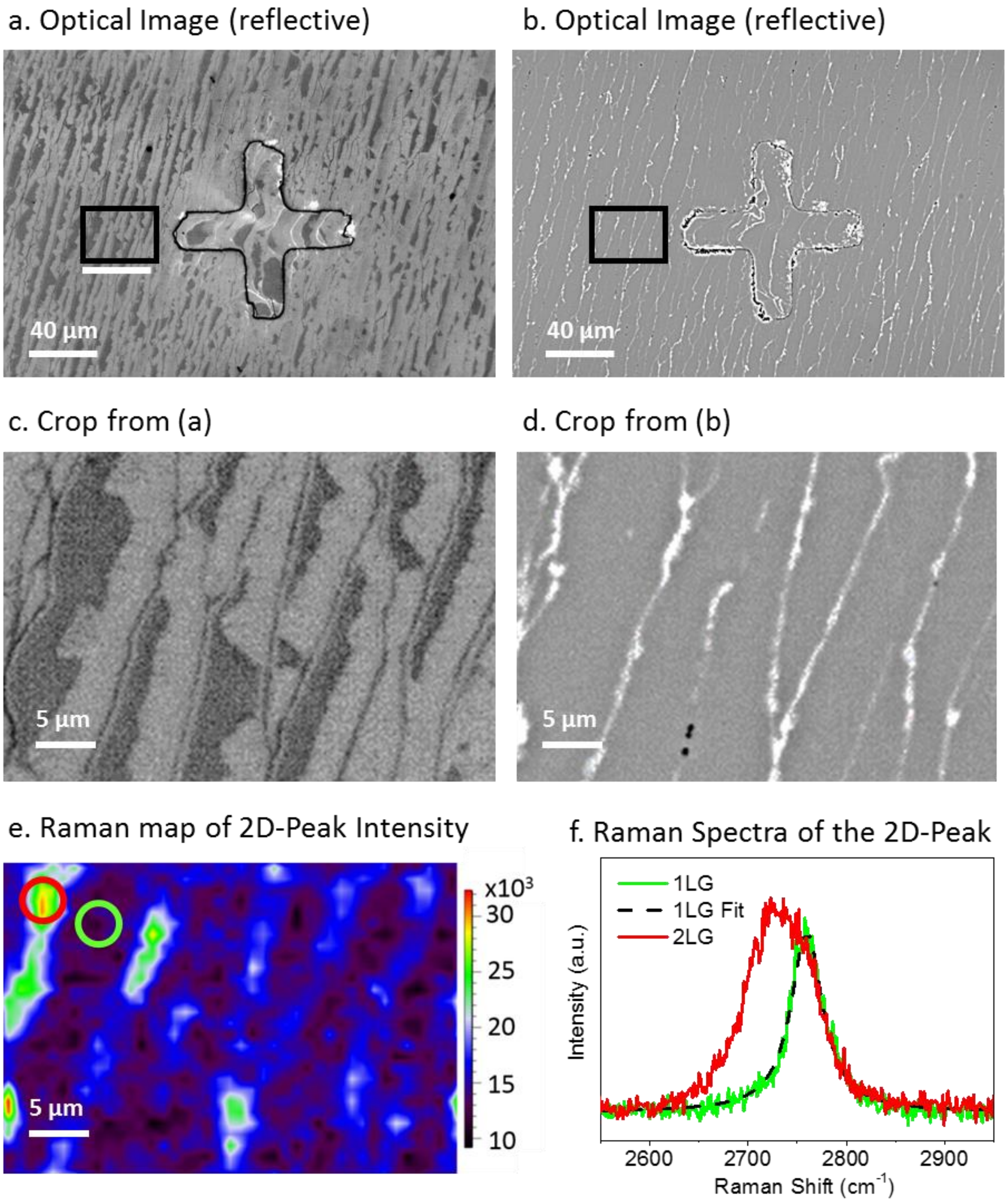

Supplementary Figure 7. Optical contrast features of EG. (a) Conventional reflective optical image of a face-to-graphite sample with partial graphene coverage. (b) Conventional reflective optical image of the same sample after a succeeding growth showing continuous background with narrow lines of higher brightness. (c) and (d) Cropped images of the region marked by the black boxes in (a) and (b) respectively. (e) 2D-Peak intensity Raman map of the same region as

in (d) after the second growth. (f) Raman spectra for 1LG and 2LG from the spots marked by green and red circles in (e), respectively. The black dot line is the Lorentzian fitting of the green curve. Raman data was acquired with 514.5 nm excitation.

Supplementary Note 6. NOTES ON THE REFLECTED INTENSITY FROM IFL, 1LG AND 2LG

We have found that the sharpness level (Supplementary Figure 8) in the advanced settings for the CLSM will strongly affect the reflected intensity due to the backstage algorithm. As suggested by the Olympus specialist, we turned off the contrast and sharpness enhancement when estimating the ratio of reflected intensity from IFL, 1LG and 2LG. We have universally observed that the reflected intensity from 1LG is ~3 % higher than that from IFL region, and the reflected intensity from 2LG is ~2 % higher than that from 1LG.

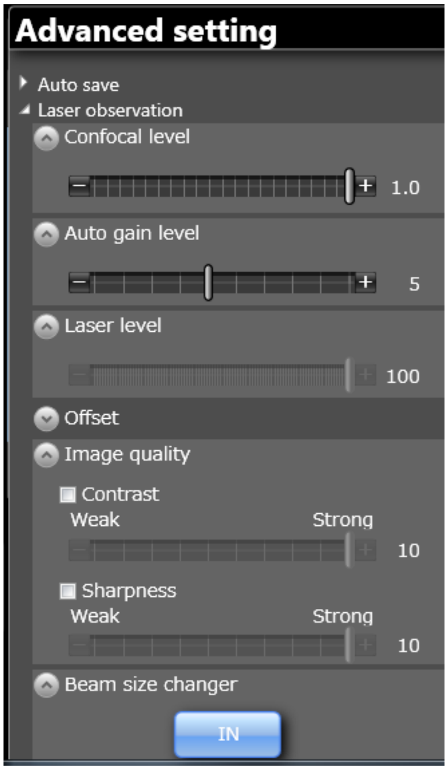

Supplementary Figure 8. CLSM advanced setting used for estimation of the change of reflected intensity from IFL, 1LG and 2LG.

Supplementary Note 7. GRAPHENE CHARACTERIZATION BY CLSM IMAGING

Since the graphene nanoribbons as well as the 2LG and FLG patches are usually submicron sized, a single CLSM scan by 20× objective and higher magnification cannot distinguish such features properly and are not suitable for the characterization of EG region larger than hundreds of micrometers. Wafer-scale EG can be characterized by stitching arrays of CLSM images scanned by 50× or 100× objective as shown in Supplementary Figure 9 and Supplementary Figure 10. Supplementary Figure 9a shows a high resolution CLSM image (produced from 64 CLSM scans by digital stitching) of a homogeneous monolayer graphene area (463 µm by 463 µm) that includes less than 1% of multilayer graphene (the irregular brighter patches). Supplementary Figures 9b, 9c, and 9d are the three zoomed-in grid CLSM images for locations marked by red boxes 1, 2 and 3 in Supplementary Figure 9a. Hall bar devices of 400 µm width fabricated from such graphene can maintain the quantum Hall effect with precise metrological accuracy up to 4 K.

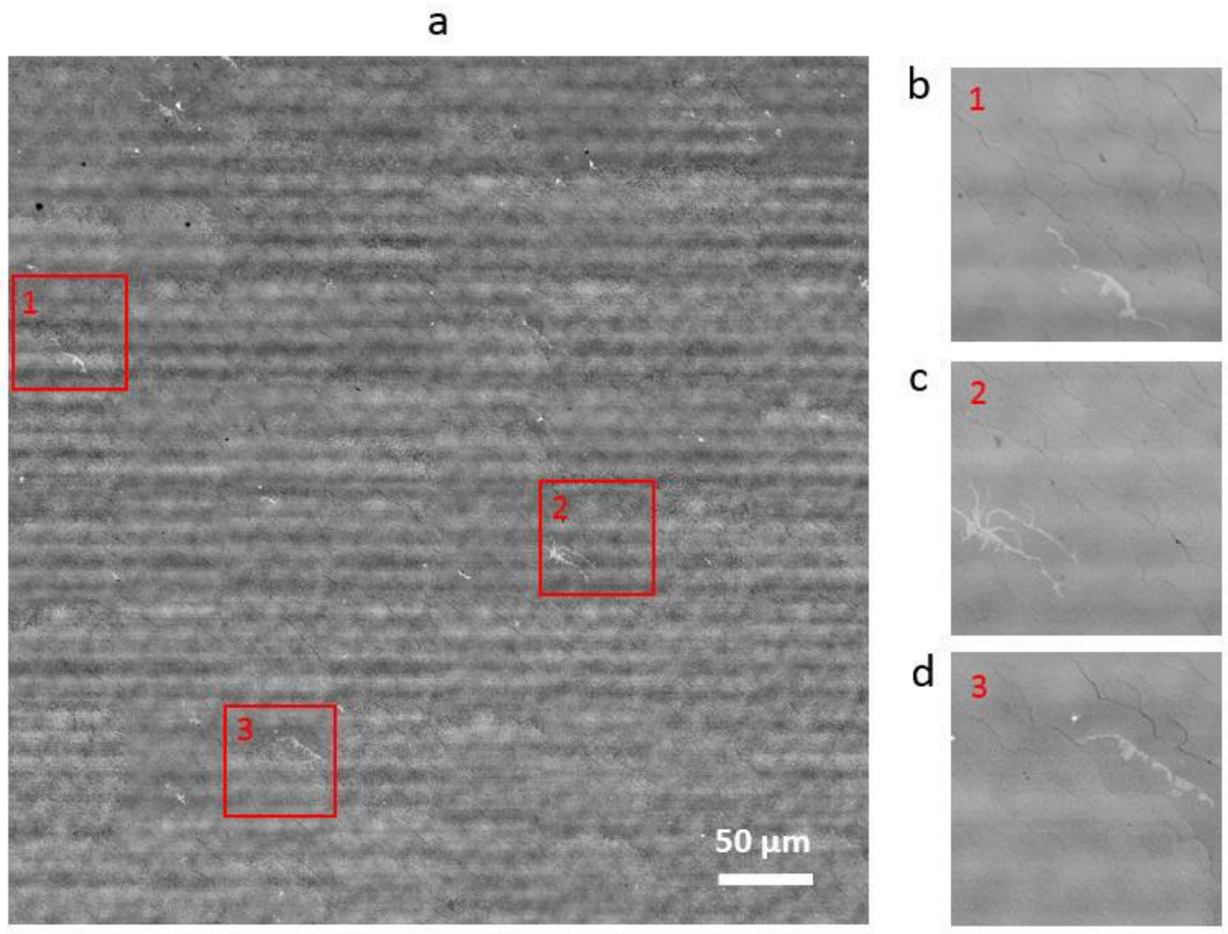

Supplementary Figure 9. (a) Stitched CLSM image of a highly uniform area of monolayer graphene with only few bilayer patches as shown in the right panels. (b)-(d) Zoom-in of the region indicated by the red boxes 1-3 in (a), respectively.

Supplementary Figure 10 shows a composite image produced in ~20 minutes from 16 CSLM scans by digital stitching. The black strip that appears in the lower region of this image is the edge of the sample. The fiducial mark (V20) is used for sample identification, and is etched into the SiC before EG is grown. Face-to-graphite growth usually produce very thick graphene layers[3] close to the edge of the sample (region 3 with much higher brightness in Supplementary Figure 10). About few hundreds of micrometers away from the edge, bilayer and few layer

patches decrease dramatically in region 2. Continuous EG with less than 1% of bilayer or few layer patches in region 1 is suitable for fabrication of quantum Hall resistance standards[2].

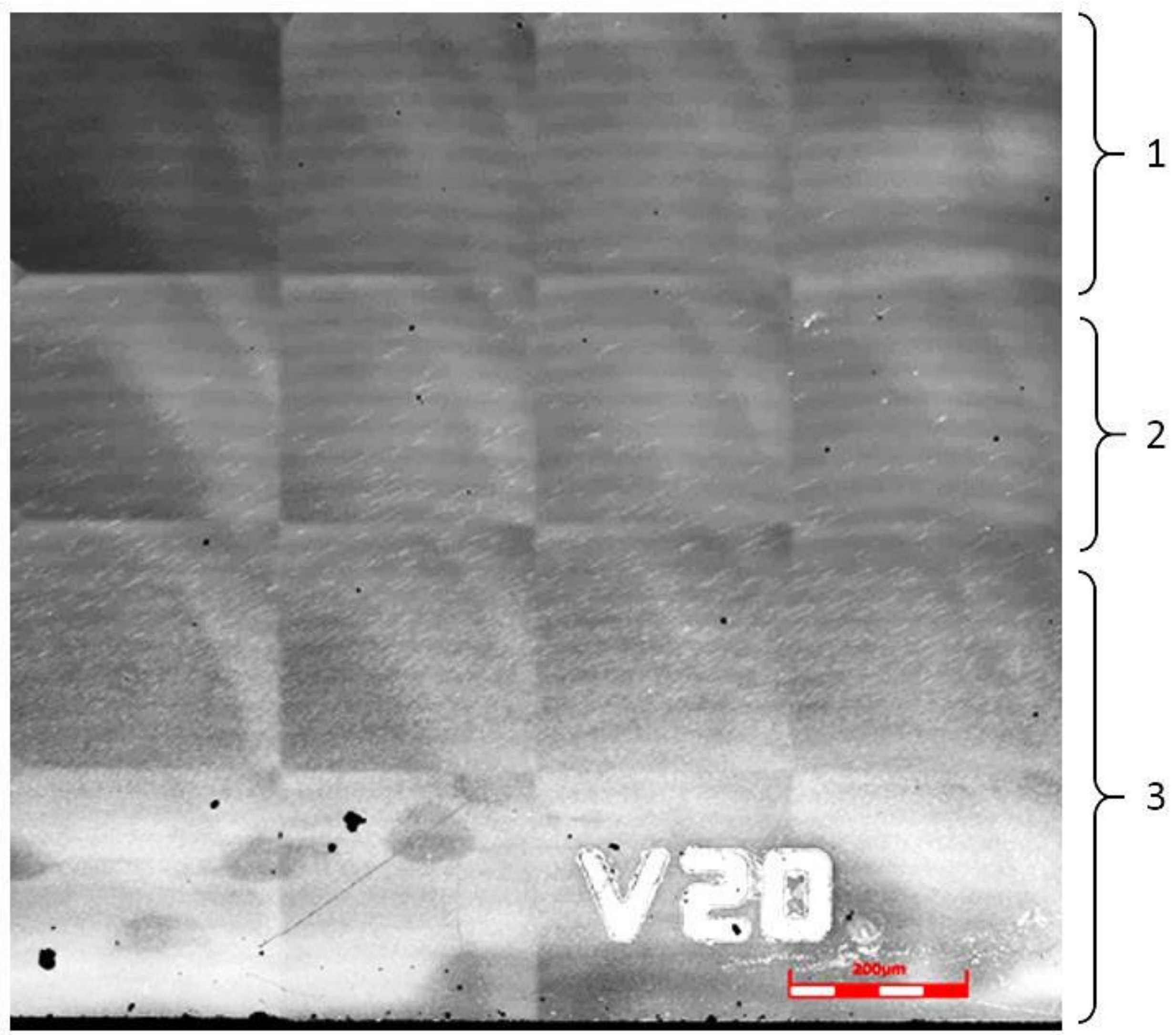

Supplementary Figure 10. Stitched CLSM image of an area near the edge of a primarily monolayer EG sample, where thicker graphene patches (bright contrast) can only be seen near the edge. Region 1 is covered by uniform and continuous 1LG. Region 2 shows increasing bilayer and few layer patches (with higher brightness). Region 3 is covered by very thick graphene.

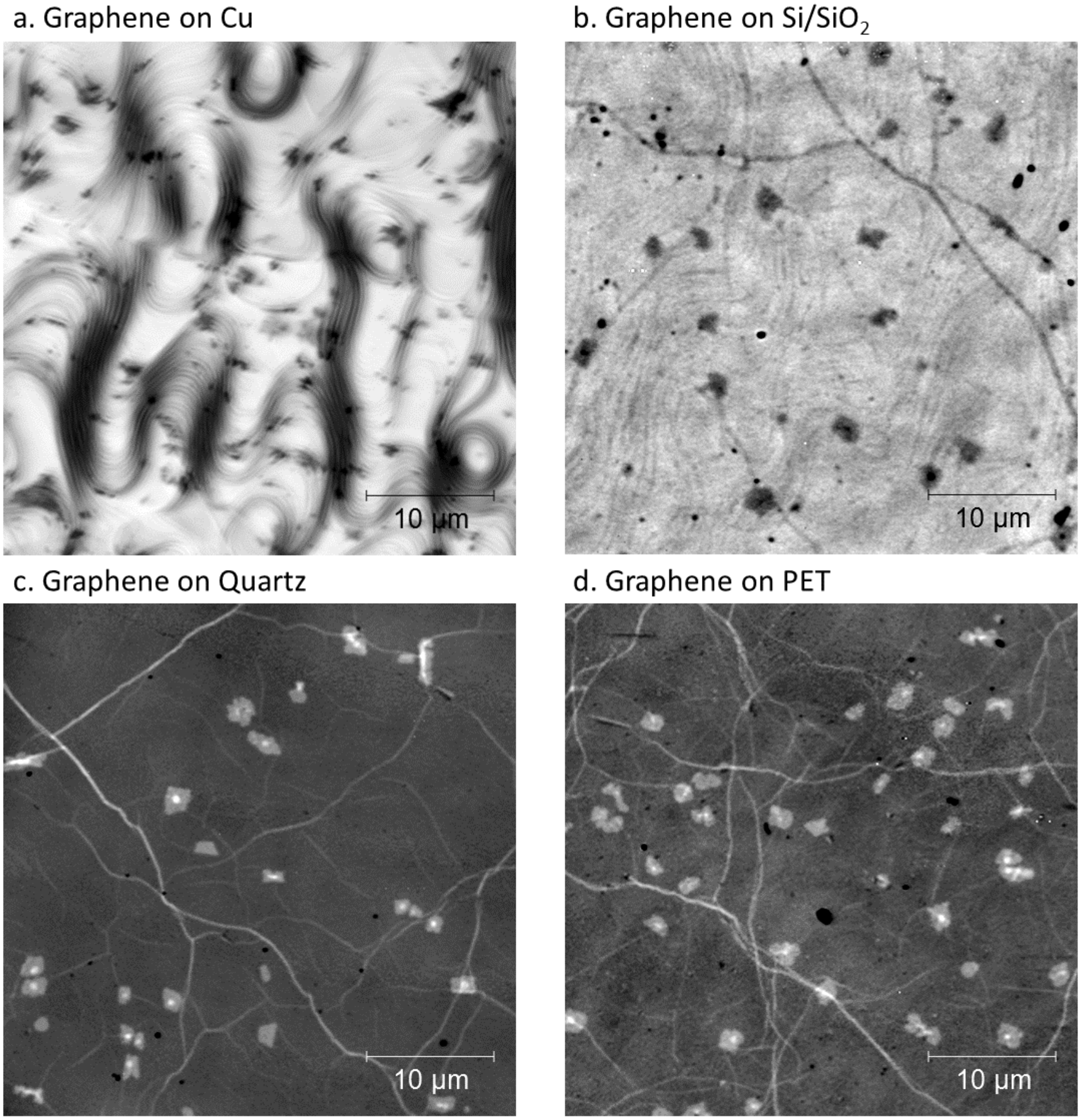

Supplementary Figure 11. CLSM imaging of CVD single layer graphene (a) grown on Cu and transferred to (b) Si/SiO_2 of 300 nm thickness, (c) quartz and (d) PET substrates. The darker islands in (a) and (b) are thicker layers of graphene and the darker lines in (b) are wrinkles. In (c) and (d), the brighter islands are thicker layers of graphene and brighter lines are wrinkles. Here,

the contrast is inverted due to the lower reflectivity of quartz and PET compared to Cu and SiO_2 at 405 nm wavelength. All samples are courtesy of Graphenea Inc.

Supplementary Note 8. DEVICE INSPECTION BY CLSM

Charge carrier mobility of graphene is an important electronic property that is usually measured using the Hall effect. However, the mobility of epitaxial graphene is strongly affected by its carrier density. To compare the quality of two graphene devices, one needs to compare the curves of mobility as a function of carrier density obtained at low temperature, as shown in Supplementary Figure 11. Here we correlate the mobility characteristic curves to the CLSM images of corresponding devices. The CLSM image (left inset in Supplementary Figure 11) of the high mobility device (red data in Supplementary Figure 11) shows almost complete graphene coverage with less than 10% of bilayer or interfacial layer inclusions. The CLSM image (right inset in Supplementary Figure 11) of the low mobility device (black data in Supplementary Figure 11) shows large portion of interfacial layer.

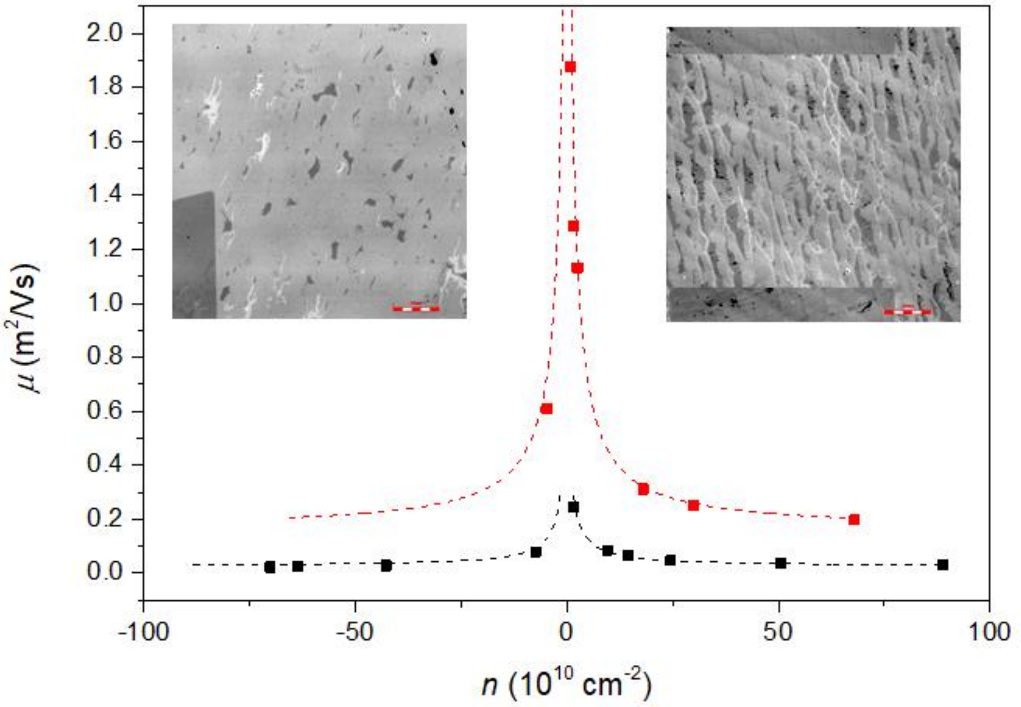

Supplementary Figure 12. Mobility of EG devices as a function of carrier density. The overall mobility of a more uniform sample (data shown in red, CLSM image in left inset) is much higher than that of another device (data shown in black, CLSM image in right inset) made from graphene area with incomplete graphene and nanoribbons.

Note: *Commercial equipment, instruments, and materials are identified in this paper in order to specify the experimental procedure adequately. Such identification is not intended to imply recommendation or endorsement by the National Institute of Standards and Technology or the United States government, nor is it intended to imply that the materials or equipment identified are necessarily the best available for the purpose.*

Supplementary References

1. Gwyddion. (2018). at <www.gwyddion.net>

2. Yang, Y., Cheng, G., Mende, P., Calizo, I. G., Feenstra, R. M., Chuang, C., Liu, C. W., Liu, C. I., Jones, G. R., Hight Walker, A. R. & Elmquist, R. E. Epitaxial graphene homogeneity and quantum Hall effect in millimeter-scale devices. *Carbon.* **115,** 229–236 (2017).
